# Circulating MicroRNAs as Biomarkers of Acute Stroke

**DOI:** 10.3390/ijms15011418

**Published:** 2014-01-20

**Authors:** Sugunavathi Sepramaniam, Jun-Rong Tan, Kay-Sin Tan, Deidre Ann DeSilva, Subramaniam Tavintharan, Fung-Peng Woon, Chee-Woon Wang, Fung-Lin Yong, Dwi-Setyowati Karolina, Prameet Kaur, Fu-Jia Liu, Kai-Ying Lim, Arunmozhiarasi Armugam, Kandiah Jeyaseelan

**Affiliations:** 1 Department of Biochemistry and Neuroscience Research Centre, Centre for Translational Medicine, Yong Loo Lin School of Medicine, National University of Singapore, 14 Medical Drive, Singapore 117599, Singapore; E-Mails: bchss@nus.edu.sg (S.S.); a0030915@nus.edu.sg (J.R.T.); bchkds@nus.edu.sg (D.S.K.); a0030101@nus.edu.sg (P.K.); l.f09@nus.edu.sg (F.J.L.); bchlimky@nus.edu.sg (K.Y.L.); bchaa@nus.edu.sg (A.A.); 2 Department of Medicine, Faculty of Medicine, University Malaya, Kuala Lumpur 50603, Malaysia; E-Mails: tanks@ummc.edu.my (K.S.T.); flyong88@yahoo.com (F.L.Y.); 3 Department of Neurology, Singapore General Hospital and National Neuroscience Institute, Singapore 169608, Singapore; E-Mails: deidre.a.de.silva@sgh.com.sg (D.A.D.); woon.fung.peng@sgh.com.sg (F.P.W.); 4 Department of Medicine and Diabetes Centre, Khoo Teck Puat Hospital, 90 Yishun Central, Singapore 768828, Singapore; E-Mail: subramaniam.tavintharan@alexandrahealth.com.sg; 5 Department of Biochemistry, Faculty of Medicine, MAHSA University College, Kuala Lumpur 59100, Malaysia; E-Mail: wang.chee@mahsa.edu.my; 6 Department of Anatomy and Developmental Biology, School of Biomedical Sciences, Faculty of Medicine, Nursing and Health Sciences, Monash University, Clayton, Victoria 3800, Australia

**Keywords:** microRNAs, cerebral ischemia, diagnosis, brain, blood, microarray

## Abstract

MicroRNAs have been identified as key regulators of gene expression and thus their potential in disease diagnostics, prognosis and therapy is being actively pursued. Deregulation of microRNAs in cerebral pathogenesis has been reported to a limited extent in both animal models and human. Due to the complexity of the pathology, identifying stroke specific microRNAs has been a challenge. This study shows that microRNA profiles reflect not only the temporal progression of stroke but also the specific etiologies. A panel of 32 microRNAs, which could differentiate stroke etiologies during acute phase was identified and verified using a customized TaqMan Low Density Array (TLDA). Furthermore we also found 5 microRNAs, miR-125b-2*, -27a*, -422a, -488 and -627 to be consistently altered in acute stroke irrespective of age or severity or confounding metabolic complications. Differential expression of these 5 microRNAs was also observed in rat stroke models. Hence, their specificity to the stroke pathology emphasizes the possibility of developing these microRNAs into accurate and useful tools for diagnosis of stroke.

## 1. Introduction

Cerebral ischemia or stroke represents one of the leading causes of mortality and serious long-term disability worldwide with a projected increase of 24.9% (from 2010) by 2030 [1]. The complexity of the disease resulting from its multiple underlying risk factors has impeded both diagnosis and potential therapy. In the past few decades, radiological assessments such as computed tomography scans and magnetic resonance imaging have facilitated diagnosis of stroke and contributed to its management. Nevertheless, the diagnostic and prognostic powers are very often limited in stroke management, in comparison to cardiovascular ischemia [2,3]. Protein biomarkers such as C-reactive protein, interleukin-6, matrix metallopeptidase 9, vascular cell adhesion molecule 1 and intercellular adhesion molecule 1 have been suggested as additional diagnostic tools. However their specificity and ability to distinguish between acute stroke and its associated risk factors or even stroke mimics is uncertain [4]. RNA-based studies have suggested promising mRNA based biomarkers since changes in gene expression are reflected in the peripheral blood RNA of stroke patients. Blood mRNA profiles could distinguish transient ischemic attack from control samples [5] and thus serve as genomic biomarkers in ischemic stroke conditions [6] and as signatures for stroke subtypes [7].

Recent reports have demonstrated that a class of small endogenously expressed non-coding RNAs, known as microRNAs (miRNAs) [8,9] could regulate gene transcription and/or translation thus orchestrating mRNA expression [10]. miRNAs bind to their target mRNAs via partial or perfect complementarity resulting in degradation and/or translational repression of the transcript. This regulatory control enforced by miRNAs makes them intriguing candidates, for changes in their expression patterns are detected even before phenotypic projection of disease onset [11]. In renal cell carcinoma, miRNA profiling provided accurate classification of poorly differentiated tumors compared to mRNA profiles and thus enabled superior diagnosis [12]. Furthermore, changes in circulating miRNA patterns have been proposed as unique and reflective of various pathologies including cardiovascular diseases [3] for they mirror the events that occur at the site of injury [13]. Specific miRNA expression has also been shown in both brain tissue and blood following ischemic stroke [14]. Besides, circulating miRNA expression varies significantly in stroke patients as well as for the different stroke subtypes [15]. Thus, circulating miRNAs manifest the potential to be developed into ischemic stroke biomarkers. Though several groups have reported on altered expression of miRNAs during ischemic stroke [14,15], the specificity to acute stroke pathology or exclusion of confounding risk factors have not been established. These are critical factors that need to be addressed in order to identify stroke specific miRNAs with clinical potential. Hence in this study, using a larger cohort and taking into consideration the various confounding risk factors, we report specific miRNAs, with high diagnostic accuracy, that are distinctly and consistently altered in acute stroke patients. The miRNAs identified in this study hold the diagnostic potential for stroke as well as etiology differentiation.

## 2. Results and Discussion

Clinical characteristics of patients and healthy individuals enlisted in this study are given in Supplementary Table S1. Three independent cohorts of patients were used. Cohort 1, consisting of 68 stroke patients and 24 healthy individuals with a mean age range of 45.5 ± 2.07 and 39.0 ± 8.10 years, respectively was used for the discovery phase of the study. Cohort 2, consisting of 101 stroke patients, were much older (average age of 59.7 ± 1.39 years) and exhibited higher degree of stroke associated risk factors. Though it is known that miRNAs were altered in response to stroke, the implications caused by associated risk factors are often not taken into consideration. To address this limitation, our study included miRNA profiles of individuals, with different age groups and varying degrees of risk factors (Cohort 3). Cohort 3 consists of 94 patients, presenting with metabolic complications (only) without any history of stroke or related disease.

### 2.1. miRNA Profiles of Stroke Patients Reveal Temporal and Etiology Based Segregation

Three hundred and fourteen (314) miRNAs were detected upon profiling of total RNA isolated from cohort 1 patients’ peripheral blood samples. Further statistical analyses based on Benjamini-Hochberg FDR correction (*p* value < 0.05) and fold-change cut-offs (≥1.2 or ≤−1.2), resulted in 105 statistically significant miRNAs (Table 1). Quantitative PCR was performed on 10 miRNAs (*p* < 0.0001) to validate our microarray data (Supplementary Table S2). Among the 105 miRNAs, 58 were downregulated (fold change < −1.2) while 47 were upregulated (fold change >1.2; Table 1). These significantly altered miRNAs, correlated with available data on stroke or brain injury [14–16] and exhibited similar expression patterns between human [15] and rat stroke models [14,16] (Supplementary Table S3). Eighty-four (84) out of the 105 miRNAs were detected at high levels in human brain at various stages of development [17], suggesting functional roles in brain activities.

**Figure 1.**
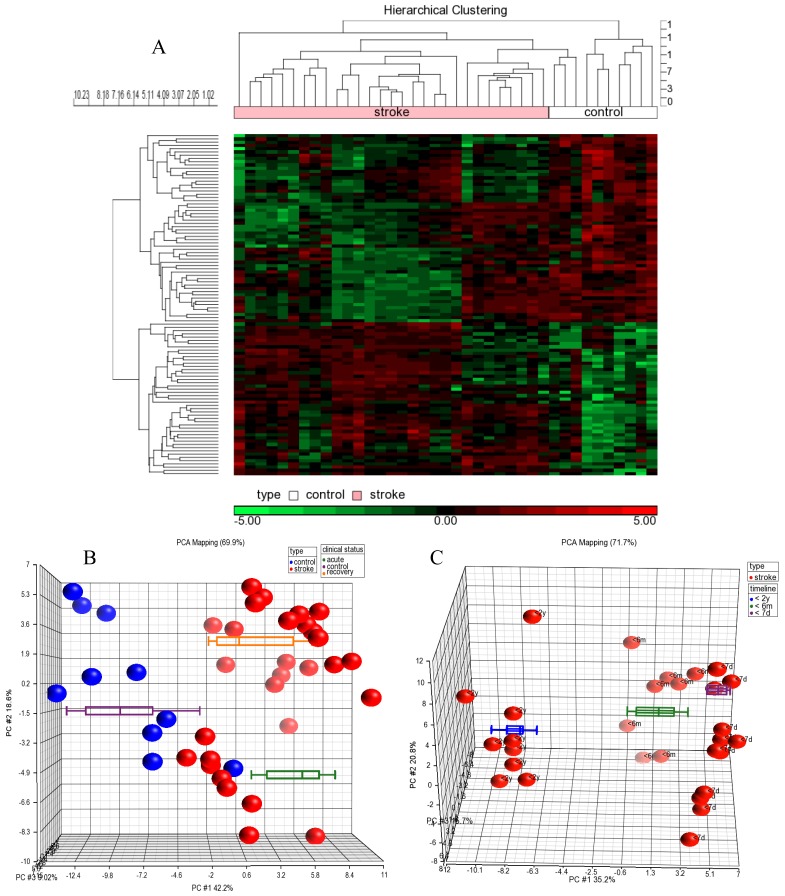
Cluster plots of miRNA profiles. (**A**) Hierarchical clustering of miRNA profiles. miRNA profiling data showed that control samples were clustered away from stroke patients. The upregulated miRNAs are shown in red and downregulated miRNAs are shown in green; (**B**) Principal Component Analysis (PCA) and Box-whisker plots. PCA analysis showed segregation of controls (blue circles) away from stroke (red circles) patients. Box-whisker plots showed that the samples segregated into three absolute categories; healthy controls (purple box-whiskers), acute stroke (green box-whiskers) and “recovered” stroke patients (orange box-whiskers); and (**C**) PCA plots with time-based segregation. Clustering of stroke samples reflected temporal evolution of miRNAs in stroke patients (purple box-whiskers < 7 days; green box-whiskers < 6 months; blue box-whiskers < 24 months).

Hierarchical clustering analyses showed that the miRNA profiles of healthy controls and stroke patients neatly assembled into two independent clusters (Figure 1A) while principal component analysis showed segregation within stroke patients (Figure 1B). Box-whisker plots showed that the segregation was due to the differences in timeline from the onset of stroke. Closer analysis revealed segregation among patients’ within 6 and 24 months of recovery (Figure 1C) and these clusters were distinct from acute patients (between day 1 to day 7) indicating temporal regulation of miRNAs during the progression of stroke pathogenesis. Nevertheless “recovered” patients clustered closer with the healthy controls suggesting a tendency for the miRNA expression, to return to normalcy.

**Figure 2.**
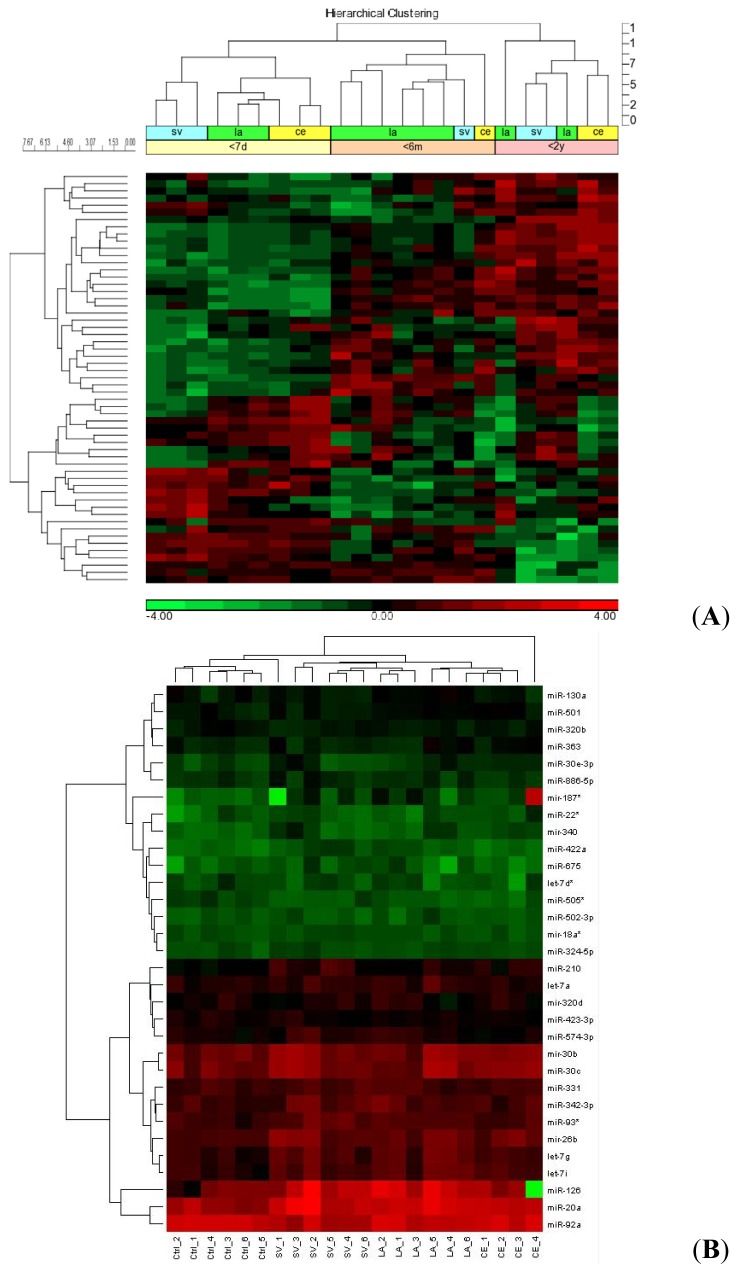
(**A**) Hierarchical clustering of miRNA profiles. Fifty seven (57) miRNAs were significantly expressed (FDR *p* value *<* 0.05) in various stroke etiologies. Hierarchical clustering identified these miRNAs as potential biomarkers of stroke etiology during the acute phase. Stroke subtypes: small vessel (SV), large artery (LA) and cardioembolic (CE). Time point from stroke onset: less than 7 days; less than 6 months; less than 2 years; and (**B**) validation of miRNA cluster. TLDA validation of the 57 miRNA cluster provided a more stringent panel of 32 miRNAs with *C*_T_ values of <32. Hierarchical clustering based on the relative expression showed that the 32 miRNAs were able to segregate the patients according to their respective stroke etiology.

Additionally when the patients were grouped based on the TOAST classification, as large artery (LA), cardioembolic (CE) and small vessel (SV), 57 miRNAs (let-7a, let-7d*, let-7g, let-7i, miR-126, -1261, -1299, -130a, -1321, -135b, -184, -187*, -18a*, -208a, -214, -20a, -22*, -26b, -26b*, -27a*, -30b, -30c, -30e*, -320b, -320d, -324-5p, -331-3p, -340, -342-3p,-361-5p, -363, -370, -381, -422a, -423-3p, -494, -501-5p, -502-3p, -505*, -525-5p, -549, -552, -553, -574-3p, -574-5p, -585, -602, -611, -617, -627, -629, -675, -7, -886-5p, -92a, -93* and -96) were identified to be significantly dysregulated among them. Hierarchical clustering revealed that these 57 miRNAs could accurately distinguish stroke subtypes within six months from stroke onset (Figure 2A). These findings were further validated on randomly selected patients (*n* = 22) using a customized TaqMan Low Density Array (TLDA). Setting cycle threshold values (*C*_T_) of 32 as a cut-off, a final panel of 32 miRNAs (let-7a, let-7d*, let-7g, let-7i, miR-126, -130a, -187*, -18a*, -20a, -22*, -26b, -30b, -30c, -30e*, -320b, -320d, -324-5p, -331-3p, -340, -342-3p, -361-5p, -363, -422a, -423-3p, -501-5p, -502-3p, -505*, -574-3p, -675, -886-5p, -92a and -93*) that could significantly distinguish the stroke etiology was obtained. Hierarchical clustering showed that this panel of miRNAs could neatly segregate the patients according to their stroke subtypes and possibly aid in the classification of stroke etiologies (Figure 2B).

### 2.2. MicroRNAs as Biomarkers of Acute Stroke

**Figure 3.**
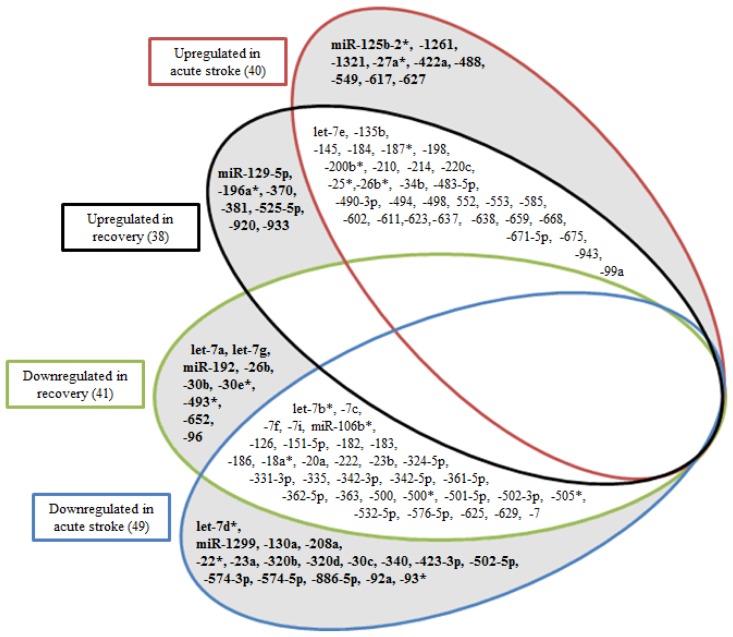
Differentially regulated miRNAs in stroke patients. miRNAs significantly altered (FDR *p <* 0.05) in acute stroke and recovery with respect to healthy controls are shown here. miRNAs unique to acute phase or recovery phase are represented in shaded regions in bold whereas those common to both categories are listed in the clear areas of the diagram.

Temporal regulation of the significantly altered 105 miRNAs suggests their importance and relevance in stroke pathology and recovery. Thus we attempted to identify those that were unique to the immediate (acute) clinical status of stroke patients. The patients were segregated into acute and recovery phase. In comparison to healthy controls, 89 and 79 miRNAs were differentially altered in acute stroke and recovered patients respectively. Of these, 63 miRNAs were common between them. Thus the remaining 26 and 16 miRNAs were unique for acute stroke and recovered patients respectively (Figure 3). let-7d*, miR-125b-2*, -1261, -1299, -130a, -1321, -208a, -22*, -23a, -27a*, -320b, -320d, -30c, -340, -422a, -423-3p, -488, -502-5p, -549a, -574-3p, -574-5p, -617, -627, -886-5p, -92a and -93* were unique for acute stroke while let-7a, let-7g, miR-129-5p, -192-5p, -196a*, -26b, -30b, -30e*, -370, -381, -493*, -525-5p, -652, -920, -933 and -96 were unique for “recovered” stroke patients (Figure 3; highlighted in bold). Among these let-7a, let-7g, miR-125b-2*, -130a, -192, -196a*, -23a, -26b, 30b, -30c, -30e*, -320b, -320d, -340, -381, -488, -652 and -92a were also reported to exhibit similar expression patterns between human [15] and rat stroke models [14,16].

Diagnostic accuracy of acute phase specific miRNAs was tested through calculating the area under curve (AUC) of receiver operating characteristic (ROC) curves, and subsequently validated in a second, independent cohort of 101 stroke patients, (Cohort 2). miR-125b-2*, -27a*, -422a, -488 and -627 showed high AUC values of 0.95, 0.89, 0.92, 0.87 and 0.84 in the Cohort 1 and 0.85, 0.86, 0.86, 0.86, and 0.76 in the Cohort 2 patients, respectively (Table 2). All five miRNAs were found to be upregulated. ROC analysis of miR-125b-2*, -27a*, -422a, -488 and -627 in the Cohort 3 patients exhibiting stroke associated risk factors only, showed poor AUC values suggesting that the selected miRNAs were indeed exclusively indicative of the onset of cerebral ischemia (Table 2). As confounding factors often serve as risk factors associated with stroke, individuals presenting with risk factors comprising of hyperlipidaemia, hypertension or diabetes (cohort 3), were included in our study. Analysis of miRNA profiles of these subjects provided a better reflection of the real pathophysiology of stroke. Incidentally, miR-145 and miR-210 had been previously reported to be potential biomarkers for stroke diagnosis [18,19]. We observed that circulating miR-145 and miR-210 were indeed significantly increased in stroke patients with strong AUC values of 0.90 and 1.0 respectively. Nevertheless the altered expression of miR-145 and miR-210 was not unique for the acute phase as we found them to be increased in recovery phase (Figure 3). miR-210 has also been proposed as a biomarker for a variety of conditions such as pancreatic and renal cell cancer as well as congestive heart failure highlighting its non-specificity and inapplicability, particularly in clinical diagnosis of stroke [20–22].

A few reports have highlighted the functional relevance of these five miRNAs with respect to brain physiology or pathology. miR-27a* is a direct target of cyclin-dependent kinase 5 (CDK5), which is predominantly expressed in the central nervous system [23]. CDK5 protein is upregulated in acute ischemia in various stroke models including post-mortem human brains [24] and plays crucial roles in neuronal survival and death. Thus upregulation of miR-27a* expression upon acute ischemia could be a defense mechanism by the cells to control the translation of CDK5 molecules. Independently, we also observed that progressive upregulation of miR-27a* correlates to neurogenesis in primary cultures of cortical neurons, thus implicating a major role for miR-27a* in neuronal regulation. Overexpression of miR-488 dysregulated corticotropin releasing hormone signalling, which is a crucial pathway activated in response to stress [25]. miR-488, suppresses the expression of panic disorder gene pro-opiomelanocortin.

**Figure 4.**
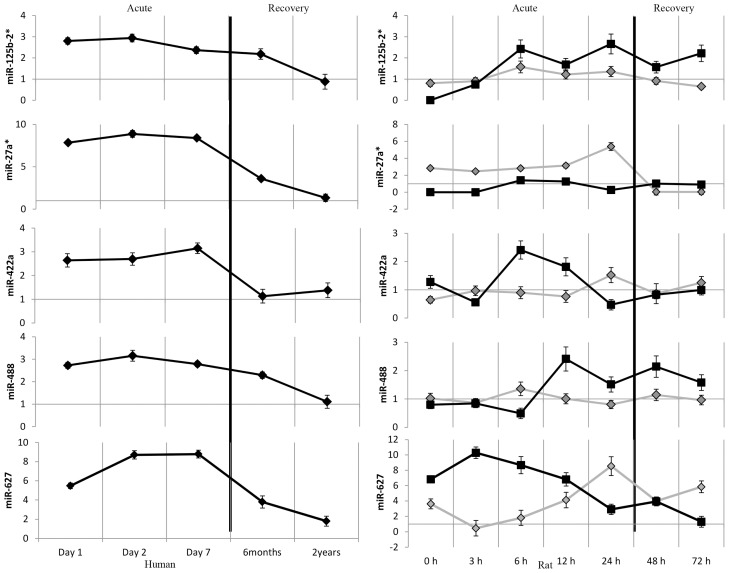
Relative miRNA expression in stroke patients and ischemic rodent models. The expression patterns of miR-125b-2*, -27a*, -422a, -488 and -627 were determined in the blood of stroke patients (*n* = 45) as well as ischemic brain and blood of rats subjected to MCA occlusion (*n* = 6). Changes in relative expression for the respective samples were determined with respect to normal healthy individuals and control rats. (black represents blood miRNA profiles; grey represents ischemic brain miRNA profiles).

Analysis of the expression profiles of miR-125b-2*, -27a*, -422a, -488 and -627, from stroke onset till recovery of two years showed that their highest expression occurred within the acute phase (one to seven days) of stroke in humans (Figure 4). To establish that their changes in expression were a consequence of the onset of stroke, an *in vivo* study using rat models was carried out. Rats subjected to Middle Cerebral Artery occlusion (MCAo) were sacrificed over a period of 0 h to 3 days. Changes in the miRNA expression in blood and brain were determined. In the brain samples, the highest expression for all the 5 miRNAs was observed within the acute phase (0 to 24 h). miR-125b-2* and miR-488 peaked at 6 h from the onset of stroke, to 1.56 ± 0.28 and 1.36 ± 0.24 fold, respectively in ischemic rat brain whereas miR-27a*, -422a and -627 peaked at 24 h from the onset of stroke, to 5.37 ± 0.46, 1.52 ± 0.28 and 8.53 ± 1.23 fold, respectively (Figure 4). The corresponding expression of these miRNAs was also at maximum levels in the rat blood, during the acute phase. miR-125b-2* blood profile showed similar expression to that of the ischemic brain, albeit with greater fold change differences. Incidentally, miR-125b-2* was shown to be conserved in the brain throughout the chimpanzee, macaque and human species, implicating its crucial functional roles in mammalian brain development [26]. In fact we also observed miR-125b-2* to have the strongest biomarker potential based on AUC values (Table 2). Except for miR-125b-2*, the remaining miRNAs exhibited an opposing profile in the brain and blood at their corresponding time points. Similar phenomenon was also observed in patients diagnosed with atherosclerotic abdominal aortic aneurysm [27]. Expression of miR-29b, -124a, -155 and -223, that were significantly increased in the atherosclerotic abdominal tissue was reduced in circulation. Similarly, miR-92a levels were found increased in acute myeloid and lymphoblastic leukemia cells, and decreased in circulation [28], suggesting complex regulatory processes occurring via the circulatory system.

Although the focus of the study was to identify specific miRNAs involved in acute stroke, we observed miR-920, a human specific miRNA, to be differentially expressed in “recovered” stroke patients. Its expression remained higher than control from the onset of stroke until day 2 and then decreased to 0.26 ± 0.752 by day 7. However, a more significant increase in miR-920 expression was observed during the recovery phase and its expression remained elevated up to 2 years after onset of stroke (*vs.* controls; Supplementary Table S4). miR-920, a target of beta-transducin repeat-containing protein (*β-TrCP*) [29] was also predicted to regulate brain-specific angiogenesis inhibitor 1 (*BAI1*). Though miR-920 has not been found in normal developing human brain [17], it may function similarly to miR-126 in atherosclerosis [30]. Zernecke *et al.* [30] showed that miR-126 targeted vascular smooth muscle cells in atherosclerotic rat models. This miRNA was in fact released from endothelial cells and circulated via apoptotic bodies to mediate its athero-protective effects on the vascular smooth muscle cells, by reducing the plaque size. Hence the possibility of human specific miR-920 being regulated in a similar manner, to cause a beneficial effect in stroke recovery needs to be further explored.

Such studies highlight the importance of miRNAs in circulation and further affirm that miRNAs found in circulation are not entirely a consequence of necrotic or apoptoic cells spilling their contents.

## 3. Experimental Section

### 3.1. Patient Enrolment (Standard Protocol Approvals, Registrations, and Patient Consents)

287 individuals (24 healthy individuals, 169 stroke patients and 94 individuals presented with metabolic syndrome) were enrolled from Khoo Teck Puat Hospital Singapore, Singapore General Hospital and University Malaya Medical Centre (UMMC), Malaysia. The study was carried out in accordance with the Declaration of Helsinki (2008) of the World Medical Association and was approved by the Medical Ethics Committee of UMMC (Ref. No: 607.20), National University of Singapore Institutional Review Board (NUS-IRB Ref. Code: 08-381, Approval: NUS-676), Ministry of Health, Singapore (MH95:03/1–11) and the Institutional Review Board (IRB) of the National University Health System and Singapore General Hospital, SingHealth (CIRB Ref. No: 2011/216/A). Ischemic stroke was confirmed through either MRI or CT imaging of the brain, and the risk factors (if any) were characterized based on the ancillary blood and routine tests [15]. Characterization of the stroke subtypes were made based on World Health Organization clinical criteria and according to Trial of Org 10172 in Acute Stroke Treatment (TOAST) classification. These patients were separated into two cohorts based on recruitment time. Cohort 1 (*n* = 68) was used for the discovery phase of the study whereas cohort 2 (*n* = 101) was used for the validation phase. Prior to blood sampling, each volunteer, gave a written informed consent. Blood was collected at the following time points following the onset of stroke: within 24 h (Day 1), within 48 h (Day 2) and within the 7 days (Day 7). Patients from outpatient clinics, in recovery phases (from 2 months to 2 years from stroke onset) were also included in this study. Cohort 3 (*n* = 98) consisting of patients exhibiting stroke-associated risk factors only, were also recruited for the study.

### 3.2. Middle Cerebral Artery Occlusion (MCAo)

Male Wistar rats (280–320 g) obtained from the Laboratory Animal Centre (National University of Singapore, Singapore) were maintained on an *ad libitum* intake of standard laboratory chow and drinking water. All animals were handled according to the Council for International Organisation of Medical Sciences on Animal Experimentation (World Health Organisation, Geneva, Switzerland) and the National University of Singapore (IACUC/NUS) guidelines for laboratory animals. A minimum number of animals (*n* = 6) were used for each category. The animals were anesthetized using 7% chlorohydrate and MCAo was induced via injection of an embolus into the middle cerebral artery [31]. Ipsilateral cerebral blood flow was measured by Laser Doppler Flowmetry (OxyFlo, Oxford-Optronix, Oxford, UK). Animals were sacrificed at 0, 3, 6, 12, 24, 48 and 72 h following MCAo and the brain samples collected were sectioned into 2-mm-thick coronal slices using an AltoSA-2160 brain-sectioning matrix (Roboz, Gaithersburg, MD, USA). The samples were stored in −80 °C until RNA processing.

### 3.3. Total RNA Isolation

Peripheral blood samples from patients and rat stroke models were collected in RNALater (Ambion, Austin, TX, USA) and stored at −80 °C until processing. Total RNA was isolated using the Ribopure™ Blood RNA isolation Kit (Ambion, Austin, TX, USA) according to manufacturer’s protocol. Total RNA from rat brain samples was extracted by a single-step method using Trizol according to the manufacturers’ protocol (Invitrogen, Carlsbad, CA, USA). RNA concentration was determined using ND-1000 Spectrophotometer (Nanodrop™, Wilmington, DE, USA). The integrity of RNA samples was verified using denaturing gel electrophoresis (15% polyacrylamide gel) and Agilent 2100 Bioanalyzer (Agilent Technologies, Santa Clara, CA, USA). Samples displaying RNA integrity number (RIN) of >7.5 were subsequently selected for microarray and quantitative PCR analysis.

### 3.4. miRNA Microarray and Statistical Analysis

miRNA profiling was performed on individual as well as pooled samples using LNA™-modified oligonucleotide (Exiqon, Vedbaek, Denmark) probes (Sanger miRBase release 12 and release 16). Peripheral blood RNA (1 μg) was 3′-end-labeled with Hy3 dye and hybridized on miRCURY LNA™ Arrays according to the manufacturer’s protocol (Exiqon, Vedbaek, Denmark). The first stage of analysis was performed using Partek^®^ 6.6 Genomics Suite software (Partek Inc., St. Louis, MO, USA). Briefly, background-subtracted median signal intensity of 100 was selected as a threshold value for inclusion of significantly detected miRNAs. Global sample variability was assessed by principal components analysis (PCA). First stage of normalization was carried out against a group of endogenous controls and the spike-in controls for each chip to avoid technical and experimental variations among the healthy and stroke samples. The normalized signal intensity value was log 2 transformed and differentially regulated (stroke *versus* healthy controls) miRNAs were selected. List of miRNAs with an absolute fold change ≥1.2 and ≤−1.2 and *p* value *<* 0.05 after the Benjamini-Hochberg false discovery rate (FDR) correction following multiple comparisons were considered significant. All statistical analyses were performed using the statistical tools provided by Partek^®^ 6.6 Genomics Suite software (Partek Inc., St. Louis, MO, USA). Receiver operating characteristic (ROC) curve values were generated using the online PSPP software (Free Software Foundation, Boston, MA, USA). Log 2 transformed normalized signal intensity ratios were used for the analysis.

### 3.5. Quantitative PCR

Validation of miRNAs was carried out using TaqMan quantitative Real-Time PCR. Briefly, 10 ng of total RNA was reverse transcribed (in 15 μL) using specific stem-loop primers. For the PCR reaction, 1.33 μL (~0.891 ng) of RT-product was used. PCR was carried out using the Applied Biosystems 7900 high throughput sequence detection system (Applied Biosystems, Foster City, CA, USA). Both RT- and PCR-reactions were performed in triplicate, in three separate experiments. *RNU44* was used as the housekeeping gene. For validation of the specific miRNA cluster (57 miRNAs), a customized low-density array (LDA) panel was used and the reaction performed according to the manufacturer’s protocols (Applied Biosystems, Foster City, CA, USA). Analysis of the LDA data was carried out using the DataAssist software provided by manufacturer (Applied Biosystems, Foster City, CA, USA). Global normalization of the LDA data was performed and the *p* values were adjusted according to the Benjamini-Hochberg FDR method.

## 4. Conclusions

Blood based miRNAs could provide an additional tool for an accurate analysis to assess diagnosis of stroke patients. Based on patient blood miRNA profiles, our study identified a panel of 32 miRNAs that could accurately distinguish stroke subtypes. We also found miR-125b-2*, -27a*, -422a, -488 and -627 to be consistently altered during acute stroke. Furthermore, using rat stroke models we provide evidence that changes in expression of these miRNAs in the brain in response to MCAo is reflected in their corresponding blood. This further confirms that the upregulation of expression observed for miR-125b-2*, -27a*, -422a, -488 and -627 is indeed a consequence of acute cerebral ischemia. Thus, we propose that miR-125b-2*, -27a*, -422a, -488 and -627 could reflect the onset of ischemic stroke and prove to be of diagnostic value.

## Supplementary Information



## Figures and Tables

**Table 1.** miRNAs significantly expressed in all stroke cases. A total of 105 miRNAs were identified to be significantly (FDR *p <* 0.05) expressed in all stroke patients. Of these, 47 miRNAs were upregulated while 58 miRNAs were downregulated in all stroke patients.

Significantly upregulated miRNAs in all stroke cases								

miRNA	*p-*value	Fold Change	miRNA	*p-*value	Fold Change	miRNA	*p-*value	Fold Change
hsa-let-7e	0.000	2.228	hsa-miR-26b*	0.004	3.725	hsa-miR-602	0.000	2.546
hsa-miR-125b-2*	0.007	1.795	hsa-miR-27a*	0.002	3.745	hsa-miR-611	0.002	1.815
hsa-miR-1261	0.003	2.145	hsa-miR-34b	0.003	1.534	hsa-miR-617	0.011	1.513
hsa-miR-129-5p	0.001	1.797	hsa-miR-370	0.002	6.215	hsa-miR-623	0.005	2.465
hsa-miR-1321	0.008	1.836	hsa-miR-381	0.014	1.719	hsa-miR-627	0.003	3.992
hsa-miR-135b	0.000	6.512	hsa-miR-422a	0.002	1.755	hsa-miR-637	0.002	1.737
hsa-miR-145	0.000	8.353	hsa-miR-483-5p	0.000	3.482	hsa-miR-638	0.000	2.410
hsa-miR-184	0.006	1.449	hsa-miR-488	0.006	2.124	hsa-miR-659	0.000	2.464
hsa-miR-187*	0.000	2.404	hsa-miR-490-3p	0.001	4.836	hsa-miR-668	0.003	2.541
hsa-miR-196a*	0.016	1.530	hsa-miR-494	0.000	2.916	hsa-miR-671-5p	0.000	2.907
hsa-miR-198	0.001	2.069	hsa-miR-498	0.000	2.479	hsa-miR-675	0.000	4.519
hsa-miR-200b*	0.000	2.573	hsa-miR-525-5p	0.002	1.999	hsa-miR-920	0.005	3.482
hsa-miR-210	0.000	4.923	hsa-miR-549	0.007	1.514	hsa-miR-933	0.015	1.437
hsa-miR-214	0.005	1.666	hsa-miR-552	0.000	4.077	hsa-miR-943	0.000	2.976
hsa-miR-220c	0.000	11.799	hsa-miR-553	0.000	15.392	hsa-miR-99a	0.000	6.401
hsa-miR-25*	0.000	2.145	hsa-miR-585	0.000	7.330			

Significantly downregulated miRNAs in all stroke cases								

miRNA	*p-*value	Fold Change	miRNA	*p-*value	Fold Change	miRNA	*p-*value	Fold Change
hsa-let-7a	0.002	−1.622	hsa-miR-222	0.003	−1.569	hsa-miR-500	0.004	−1.626
hsa-let-7b*	0.001	−2.087	hsa-miR-23a	0.008	−1.730	hsa-miR-500*	0.000	−2.309
hsa-let-7c	0.005	−1.663	hsa-miR-23b	0.003	−1.380	hsa-miR-501-5p	0.000	−2.450
hsa-let-7d*	0.002	−3.050	hsa-miR-26b	0.003	−1.563	hsa-miR-502-5p	0.001	−2.112
hsa-let-7f	0.001	−1.790	hsa-miR-30b	0.003	−1.503	hsa-miR-502-3p	0.000	−2.264
hsa-let-7g	0.007	−1.578	hsa-miR-30c	0.011	−1.442	hsa-miR-505*	0.000	−2.356
hsa-let-7i	0.005	−1.703	hsa-miR-30e*	0.001	−2.534	hsa-miR-532-5p	0.000	−1.775
hsa-miR-106b*	0.009	−1.332	hsa-miR-320b	0.016	−2.085	hsa-miR-574-5p	0.005	−1.569
hsa-miR-126	0.000	−2.041	hsa-miR-320d	0.016	−1.766	hsa-miR-574-3p	0.014	−1.528
hsa-miR-1299	0.008	−1.740	hsa-miR-324-5p	0.000	−2.499	hsa-miR-576-5p	0.000	−2.657
hsa-miR-130a	0.006	−1.486	hsa-miR-331-3p	0.000	−1.523	hsa-miR-625	0.000	−2.289
hsa-miR-151-5p	0.001	−1.883	hsa-miR-335	0.002	−2.142	hsa-miR-629	0.000	−1.908
hsa-miR-18a*	0.000	−1.647	hsa-miR-340	0.007	−1.498	hsa-miR-652	0.001	−1.398
hsa-miR-182	0.000	−2.533	hsa-miR-342-3p	0.000	−2.066	hsa-miR-7	0.000	−2.229
hsa-miR-183	0.000	−2.137	hsa-miR-342-5p	0.000	−2.135	hsa-miR-886-5p	0.006	−2.427
hsa-miR-186	0.002	−1.793	hsa-miR-361-5p	0.000	−2.022	hsa-miR-92a	0.003	−1.420
hsa-miR-192	0.002	−2.183	hsa-miR-362-5p	0.002	−1.944	hsa-miR-93*	0.002	−1.632
hsa-miR-20a	0.003	−1.633	hsa-miR-363	0.000	−1.769	hsa-miR-96	0.005	−2.101
hsa-miR-208a	0.005	−2.085	hsa-miR-423-3p	0.016	−1.614			
hsa-miR-22*	0.011	−1.441	hsa-miR-493*	0.011	−2.058			

**Table 2.** Receiver operating characteristic (ROC) curve analysis. Values for area under the ROC curve (AUC) for selected miRNAs with biomarker potential as well as their 95% confidence intervals (CI) are listed.

miRNAs	Cohort 1		Cohort 2		Cohort 3	
	Stroke patients		Stroke patients		Metabolic syndrome patients	

	AUC	95% CI	AUC	95% CI	AUC	95% CI
miR-125-2*	0.95 ± 0.04	0.89–1.02	0.85 ± 0.05	0.77–0.93	0.67 ± 0.19	0.36–0.98
miR-27a*	0.89 ± 0.07	0.77–1.01	0.88 ± 0.05	0.81–0.96	0.67 ± 0.16	0.40–0.93
miR-422a	0.92 ± 0.06	0.82–1.02	0.86 ± 0.07	0.75–0.97	0.30 ± 0.17	0.02–0.58
miR-488	0.87 ± 0.08	0.75–1.00	0.86 ± 0.06	0.72–0.92	0.56 ± 0.21	0.20–0.91
miR-627	0.84 ± 0.08	0.70–0.98	0.76 ± 0.06	0.66–0.87	0.41 ± 0.25	0.01–0.82
miR-920	0.81 ± 0.05	0.68–0.94	1.00 ± 0.00	1.00–1.00	0.33 ± 0.16	0.07–0.60
