# Supplementary material for: Circulating MicroRNAs as Biomarkers of Acute Stroke
Source: Int J Mol Sci. 2014 Jan 20;15(1):1418–32. doi: 10.3390/ijms15011418 (PMC3907877; doi:10.3390/ijms15011418)
Supplement: Supplementary file 1 [file ijms-15-01418-s001.pdf]

Supplementary Information

Table S1. Demographic variables of individuals recruited for this study.

	Healthy controls (*n* = 24)	Cohort 1 Stroke Patients (*n* = 68)	Cohort 2 Stroke Patients (*n* = 101)	Cohort 3 Metabolic Syndrome Patients (*n* = 94)
**Age (Years ± SEM)**	39.0 ± 8.10	45.5 ± 2.07	59.7 ± 1.39	39.4 ± 9.15
		**Gender**		
**Female, n (%)**	6 (25.0)	28 (41.14)	39 (38.61)	27 (28.72)
**Male, n (%)**	18 (75.0)	40 (58.82)	62 (61.39)	67 (71.28)
**Ethnicity**	Asian	Asian	Asian	Asian
		**Risk Factors, n (%)**		
**Hypertension**	NA	41(63.08)	78 (77.59)	28 (29.78)
**Diabetes**	NA	27(41.54)	45 (44.55)	31 (33.00)
**Hyperlipidaemia**	NA	31(47.69)	76 (75.24)	74 (78.72)

Table S2. Validation of miRNA expression in all stroke samples. Expression of 10 highly significant miRNAs ($p < 0.0001$) were validated using qPCR. Expression of the miRNAs was determined in all stroke samples (regardless of timepoints) as well as samples clustered according to their timepoints. Data is represented as a relative expression with respect to healthy controls ± SEM.

miRNAs	All Stroke	Acute Stroke			Recovery	
		**Day 1**	**Day 2**	**Day 7**	**<6 months**	**<2 years**
**miR-135b**	3.62 ± 1.86	4.19 ± 1.50	3.59 ± 2.49	2.69 ± 1.72	2.86 ± 2.68	2.79 ± 1.89
**miR-145**	1.65 ± 0.70	1.25 ± 0.80	1.30 ± 0.93	1.49 ± 0.93	1.89 ± 0.57	1.13 ± 0.15
**miR-210**	2.07 ± 1.36	2.29 ± 1.54	2.55 ± 2.01	1.44 ± 0.79	1.08 ± 0.79	1.66 ± 1.31
**miR-494**	1.74 ± 0.76	1.39 ± 0.42	2.35 ± 3.12	0.99 ± 0.60	1.25 ± 0.99	2.14 ± 2.40
**miR-498**	4.19 ± 2.51	4.44 ± 3.57	4.11 ± 2.79	1.38 ± 1.19	2.18 ± 2.87	5.85 ± 6.59
**miR-126**	0.81 ± 0.52	0.95 ± 0.90	0.80 ± 0.25	0.67 ± 0.54	0.61 ± 0.53	0.57 ± 0.49
**miR-182**	0.87 ± 0.57	0.61 ± 0.13	0.68 ± 0.44	0.73 ± 0.52	0.62 ± 0.57	0.55 ± 0.47
**miR-183**	0.56 ± 0.36	0.62 ± 0.48	0.46 ± 0.43	0.38 ± 0.25	0.30 ± 0.20	0.41 ± 0.36
**miR-342-3p**	0.63 ± 0.35	0.64 ± 0.20	0.58 ± 0.40	0.36 ± 0.21	0.63 ± 0.55	0.74 ± 0.62
**miR-361-5p**	0.68 ± 0.28	0.73 ± 0.22	0.60 ± 0.31	0.53 ± 0.26	0.56 ± 0.48	0.67 ± 0.56

Table S3. Circulating miRNAs identified in stroke and stroke-related co-morbidities. With respect to our 105 miRNAs cluster, several studies reporting on miRNAs implicated in the pathology of stroke and its risk factors are compiled in this table. Among the 105 miRNAs, those highlighted in bold exhibit similar expression profiles when compared to reports involving rat stroke models.

**miRNAs**	**Fold change**	**Tan *et al.* (2009)**	**Jeyaseelan *et al.* (2008)**		**Liu *et al.* (2010)**		**Dharap *et al.* (2009)**	**Lim *et al.* (2010)**	**Origin**	**Circulating miRNA (serum/plasma) expression in stroke co-morbidity**
		**Human**	**Rat**		**Rat**		**Rat**	**Rat**		
		**Blood (2 years)**	**Blood (24 h)**	**Brain (24 h)**	**Blood (24 h)**	**Brain (24 h)**	**Brain (24 h)**	**Brain (24 h)**		
**let-7a**	−1.62		↓	↓	↓	ND		↓		
let-7b*	−2.09									
**let-7c**	−1.66				↓	ND				
let-7d*	−3.05									
let-7e	2.23	↑								
**let-7f**	−1.79	↓	↓	↓						
**let-7g**	−1.58	↓			↓	ND				
**let-7i**	−1.70	↓	↓	↓						
miR-106b*	−1.33									
miR-125b-2*	1.80							↑		
**miR-126**	−2.04	↓			↓	ND		↓	Endothelial cell	↓ Diabetes & Atherosclerosis
miR-1261	2.14	↑								
miR-129-5p	1.80	↑						↑		
miR-1299	−1.74									
**miR-130a**	−1.49				↓	ND			Bone marrow	↓ Megakaryocytopoiesis
miR-1321	1.84									
miR-135b	6.51							↑		
**miR-145**	8.35						↑		Smooth muscle	↑Stroke<br>↓ Atherosclerosis
miR-151-5p	−1.88									
miR-182	−2.53									
miR-183	−2.14									

Table S3. *Cont.*

miRNAs	Fold change	Tan *et al.* (2009)	Jeyaseelan *et al.* (2008)		Liu *et al.* (2010)		Dharap *et al.* (2009)	Lim *et al.* (2010)	Origin	Circulating miRNA (serum/plasma) expression in stroke co-morbidity
		Human	Rat		Rat		Rat	Rat		
		Blood (2 years)	Blood (24 h)	Brain (24 h)	Blood (24 h)	Brain (24 h)	Brain (24 h)	Brain (24 h)		
**miR-184**	1.45	↑						↑		
miR-186	−1.79	↓								
miR-187*	2.40									
miR-18a*	−1.65									
**miR-192**	−2.18				↓	ND				
**miR-196a***	1.53							↑		
miR-198	2.07									
miR-200b*	2.57									
miR-208a	−2.08								Heart	↑ Myocardial Infarction
**miR-20a**	−1.63	↓			↓	ND				
**miR-210**	4.92	ND	ND	↑	↓	↓	ND	↓	Brain	
**miR-214**	1.67						↑	↑		
miR-22*	−1.44									
miR-220c	11.80									
**miR-222**	−1.57				↓	ND			Endothelial cell	
**miR-23a**	−1.73		↓	↓						
**miR-23b**	−1.38				↓	ND				
**miR-25***	2.14	↑						↑		
**miR-26b**	−1.56	↓			↓	ND	↓			
**miR-26b***	3.72							↑		
miR-27a*	3.74									
**miR-30b**	−1.50	↓			↓	ND				
**miR-30c**	−1.44				↓	ND		↓	Bone marrow	↑ Megakaryocytopoiesis
miR-30e*	−2.53									

Table S3. *Cont.*

**miRNAs**	**Fold change**	**Tan *et al.* (2009)**	**Jeyaseelan *et al.* (2008)**		**Liu *et al.* (2010)**		**Dharap *et al.* (2009)**	**Lim *et al.* (2010)**	**Origin**	**Circulating miRNA (serum/plasma) expression in stroke co-morbidity**
		**Human**	**Rat**		**Rat**		**Rat**	**Rat**		
		**Blood (2 years)**	**Blood (24 h)**	**Brain (24 h)**	**Blood (24 h)**	**Brain (24 h)**	**Brain (24 h)**	**Brain (24 h)**		
**miR-320b**	−2.08		↓	↑	↓	ND			Endothelial cell	↓ Diabetes
**miR-320d**	−1.77		↓	↑	↓	ND			Endothelial cell	↓ Diabetes
miR-324-5p	−2.50									
**miR-331-3p**	−1.52				↓	ND				
**miR-335**	−2.14						↓			
**miR-340**	−1.50				↓	ND				
miR-342-3p	−2.07									
**miR-342-5p**	−2.13				↓	ND		↓		
**miR-34b**	1.53	↑	ND	ND	ND	ND	ND	↑	Brain	
miR-361-5p	−2.02								Heart	↑ Heart failure
miR-362-5p	−1.94									
**miR-363**	−1.77							↓		
miR-370	6.21									
miR-381	1.72							↑		
miR-422a	1.75									
miR-423-3p	−1.61									
**miR-483-5p**	2.70							↑		
**miR-488**	2.12							↑		
miR-490-3p	4.84									
miR-493*	−2.06									
**miR-494**	2.92	↑	↑	↑				↑		
miR-498	2.48									
**miR-500**	−1.63				↓	↑				
miR-500*	−2.31									

Table S3. *Cont.*

**miRNAs**	**Fold change**	**Tan *et al.* (2009)**	**Jeyaseelan *et al.* (2008)**		**Liu *et al.* (2010)**		**Dharap *et al.* (2009)**	**Lim *et al.* (2010)**	**Origin**	**Circulating miRNA (serum/plasma) expression in stroke co-morbidity**
		**Human**	**Rat**		**Rat**		**Rat**	**Rat**		
		**Blood (2 years)**	**Blood (24 h)**	**Brain (24 h)**	**Blood (24 h)**	**Brain (24 h)**	**Brain (24 h)**	**Brain (24 h)**		
**miR-501-5p**	−2.45							↓		
miR-502-3p	−2.26									
miR-502-5p	−2.11									
miR-505*	−2.36									
miR-525-5p	2.00									
**miR-532-5p**	−1.78				↓	ND				
miR-549	1.51									
miR-552	4.08	ND	ND	ND	ND	ND	ND	ND	Brain[73]	
miR-553	15.39									
miR-574-3p	−1.53									
miR-574-5p	−1.57									
miR-576-5p	−2.66									
miR-585	7.33									
miR-602	2.55	↑								
miR-611	1.82									
miR-617	1.51									
miR-623	2.46									
miR-625	−2.29									
miR-627	3.99									
miR-629	−1.91									
miR-637	1.74	↑								
miR-638	2.41	↑								
**miR-652**	−1.40				↓	ND				
miR-659	2.46									

Table S3. *Cont.*

**miRNAs**	**Fold change**	**Tan *et al.* (2009)**	**Jeyaseelan *et al.* (2008)**		**Liu *et al.* (2010)**		**Dharap *et al.* (2009)**	**Lim *et al.* (2010)**	**Origin**	**Circulating miRNA (serum/plasma) expression in stroke co-morbidity**
		**Human**	**Rat**		**Rat**		**Rat**	**Rat**		
		**Blood (2 years)**	**Blood (24 h)**	**Brain (24 h)**	**Blood (24 h)**	**Brain (24 h)**	**Brain (24 h)**	**Brain (24 h)**		
miR-668	2.54	↑							Heart	
**miR-671-5p**	2.91							↑		
miR-675	4.52									
**miR-7**	−2.23	↓	ND	↓	↑	ND	↓	↓	Brain	
miR-886-5p	−2.43									
miR-920	3.48									
**miR-92a**	−1.42				↓	ND		↓	Endothelial cell	↓ Atherosclerosis
miR-93*	−1.63									
miR-933	1.44									
miR-943	2.98									
miR-96	−2.10								Platelet	↓Coagulation
**miR-99a**	6.40							↑		

Table S4. Expression of human specific miR-920. Expression of miR-920 was determined in all stroke samples across various time points. Data is represented as a relative expression with respect to healthy controls ± SEM.

**miRNA**	**Acute Stroke**			**Recovery**	
	**Day 1**	**Day 2**	**Day 7**	**<6 months**	**<2 years**
**miR-920**	2.20 ± 0.640	1.82 ±1.196	0.26 ± 0.752	2.70 ± 0.551	1.50 ± 0.205

© 2014 by the authors; licensee MDPI, Basel, Switzerland. This article is an open access article distributed under the terms and conditions of the Creative Commons Attribution license (http://creativecommons.org/licenses/by/3.0/).
